# Mitochondrial Ribosome Dysfunction in Human Alveolar Type II Cells in Emphysema

**DOI:** 10.3390/biomedicines10071497

**Published:** 2022-06-24

**Authors:** Loukmane Karim, Chih-Ru Lin, Beata Kosmider, Gerard Criner, Nathaniel Marchetti, Sudhir Bolla, Russell Bowler, Karim Bahmed

**Affiliations:** 1 Department of Microbiology, Immunology, and Inflammation, Temple University, Philadelphia, PA 19140, USA; loukmane.karim@temple.edu (L.K.); chih-ru.lin@temple.edu (C.-R.L.); beata.kosmider@temple.edu (B.K.); 2 Center for Inflammation and Lung Research, Temple University, Philadelphia, PA 19140, USA; 3 Department of Thoracic Medicine and Surgery, Temple University, Philadelphia, PA 19140, USA; gerard.criner@tuhs.temple.edu (G.C.); nathaniel.marchetti@tuhs.temple.edu (N.M.); sudhir.bolla@tuhs.temple.edu (S.B.); 4 Department of Medicine, National Jewish Health, Denver, CO 80206, USA; bowlerr@njhealth.org

**Keywords:** alveolar type II cells, emphysema, mitochondria, mitoribosome, lung

## Abstract

Pulmonary emphysema is characterized by airspace enlargement and the destruction of alveoli. Alveolar type II (ATII) cells are very abundant in mitochondria. OXPHOS complexes are composed of proteins encoded by the mitochondrial and nuclear genomes. Mitochondrial 12S and 16S rRNAs are required to assemble the small and large subunits of the mitoribosome, respectively. We aimed to determine the mechanism of mitoribosome dysfunction in ATII cells in emphysema. ATII cells were isolated from control nonsmokers and smokers, and emphysema patients. Mitochondrial transcription and translation were analyzed. We also determined the miRNA expression. Decreases in ND1 and UQCRC2 expression levels were found in ATII cells in emphysema. Moreover, nuclear NDUFS1 and SDHB levels increased, and mitochondrial transcribed ND1 protein expression decreased. These results suggest an impairment of the nuclear and mitochondrial stoichiometry in this disease. We also detected low levels of the mitoribosome structural protein MRPL48 in ATII cells in emphysema. Decreased 16S rRNA expression and increased 12S rRNA levels were observed. Moreover, we analyzed miR4485-3p levels in this disease. Our results suggest a negative feedback loop between miR-4485-3p and 16S rRNA. The obtained results provide molecular mechanisms of mitoribosome dysfunction in ATII cells in emphysema.

## 1. Introduction

Chronic obstructive pulmonary disease (COPD) impairs lung function and reduces lung capacity [1]. COPD involves emphysema, a progressive disease characterized by airspace enlargement and the destruction of alveoli which decreases the surface area available for gas exchange [2,3]. Cigarette smoke is the main risk factor for emphysema development; however, the exact mechanism remains unclear.

The alveolar wall comprises alveolar type II (ATII) cells and alveolar type I (ATI) cells [4,5,6]. ATII cells produce and secrete pulmonary surfactants, proliferate, and differentiate into ATI cells [6]. Exposure to cigarette smoke induces reactive oxygen species (ROS) generation and ATII cell injury [7]. Oxidative stress can trigger antioxidant imbalance, cell cycle arrest, pro-inflammatory responses, and senescence leading to cell death [8]. Most studies focused on pulmonary emphysema pathophysiology have used lung tissue and multiple cell types; however, there are very limited reports on ATII cells isolated from patients with this disease.

ATII cells are abundant in mitochondria, which produce ATP important for their high metabolism and function [9,10]. Mitochondrial dysfunction or altered morphology correlates with impaired bioenergetics and dynamics [11,12]. Additionally, this can increase ROS generation leading to mitochondrial DNA (mtDNA) damage which contributes to cell death [13,14]. The respiratory chain complexes (RCC), dedicated to the ATP-generating oxidative phosphorylation system (OXPHOS), have dual origins. There are 13 genes encoded in the mtDNA and 84 genes encoded in the nuclear DNA, and their expression is coordinated to keep a certain stoichiometry [15,16]. OXPHOS complexes are present in the inner membrane of mitochondria and are composed of complexes I-V. Additionally, mtDNA encodes 22 transfer RNAs (tRNAs) and 2 ribosomal RNAs (rRNAs). The 12S rRNA and 16S rRNA are encoded by *MT-RNR1* and *MT-RNR2*, respectively, and are required for mitoribosome assembly, along with the 78 nuclear-encoded mitochondrial proteins required to translate the mtDNA-encoded subunits [8]. PTCD1 and PTCD3 are RNA-binding proteins, which interact with and stabilize 16S rRNAs and 12S rRNA, respectively, and contribute to mitoribosome stability and mitochondrial protein translation [17,18]. MRPS27 and MRPL48 are among the components of the small and large subunits, respectively, in the mitoribosome. The effects of oxidative stress on mitochondrial transcription and translation in airway cells or skeletal muscles in COPD patients [19,20,21,22,23] and mouse models of emphysema [24] have been reported. However, mitochondrial RNA homeostasis in human ATII cells in emphysema is unknown.

miRNAs are single-stranded non-coding RNAs that target mRNAs, regulating their stability and gene expression [25,26]. Furthermore, miRNAs translocate to mitochondria or are generated in these organelles and are involved in the translation of mitochondrial transcripts [27,28]. The interaction of miRNAs with their target genes is dynamic and regulated by several factors, including miRNA abundance and miRNA–mRNA affinity. In addition, circulating miRNAs are used as biomarkers of COPD [29]. However, there are no reports on miRNA’s effect on mitochondrial transcription and translation in human primary ATII cells in emphysema.

We recently showed that ATII cells isolated from emphysema patients display mitochondrial dysfunction, a decreased mtDNA amount, and increased mtDNA damage [30]. Here, we studied mitochondrial ribosome function in ATII cells obtained from individuals with this disease.

## 2. Materials and Methods

### 2.1. ATII Cell Isolation from Human Lungs

Lungs were obtained from the Gift of Life Foundation. Nonsmokers never smoked, and smokers smoked at least 5 to 10 cigarettes per day for at least ten years. Emphysematous lungs were obtained from transplantation performed at Temple University. We used lungs from males and females, 45 to 69 years old (N = 4–14 per group). ATII cells were isolated as we previously described [30]. Plasma was obtained from nonsmokers, smokers, and COPD patients (Table 1).

### 2.2. Western Blotting

The following primary antibodies were used for Western blotting: ATP5A (sc-136178), COX4 (sc-517553), COX5A (sc-376907), UQCRC2 (sc-390378), SDHB (sc-271548), NDUFS1 (sc-271510) from Santa Cruz Biotechnology (Dallas, TX, USA). We obtained MRPS27 (66724-1-Ig), MRPL48 (14667-1-AP), ND1 (19703-1-AP), and PTCD3 (25158-1-AP) antibodies from Proteintech (Rosemont, IL, USA), PTCD1 antibody (A16219) from Abclonal (Woburn, MA, USA), β-actin (A5441) and vinculin antibodies (V9131) from Sigma-Aldrich (St. Louis, MO, USA). We used the corresponding anti-rabbit HRP and anti-mouse HRP secondary antibodies (Jackson ImmunoResearch, West Grove, PA, USA).

### 2.3. RT-PCR

RNA was isolated using Quick-RNA MiniPrep (Zymo Research, Irvine, CA, USA) and converted into cDNA using the SuperScript IV First-Strand Synthesis System (Invitrogen, Waltham, MA, USA). We used the SYBR Green Master Mix. mRNA expression was normalized to GAPDH levels and calculated using the 2^ddCt method [31] using validated primers [32] obtained from Invitrogen (Table S1).

### 2.4. miRNA Analysis

miRNAs were isolated using the miRNeasy Kit (Qiagen, Hilden, Germany) and converted into cDNA using the SuperScript IV First-Strand Synthesis System (Invitrogen) and stem-loop RT primers. The resulting cDNA with a miRNA-specific forward primer was measured to quantify the miRNA expression by RT-PCR using SYBR Green Master Mix. miRNA expression was normalized to U6 expression using the 2^ddCt method (Table S1). For miRNA overexpression, miR-4485-3p mimics or non-target (NT) (Genscript Biotech, Piscataway, NJ, USA) were used at 100 nM and incubated with Lipofectamine RNAiMAX (Invitrogen). A549 cells were analyzed 72 h post-transfection by RT-PCR as we have previously described [33].

### 2.5. Immunofluorescence

The purity of freshly isolated ATII cells was determined by immunocytofluorescence using surfactant protein-C (SP-C, sc-518029, Santa Cruz Biotechnology), p63 (051K4894, Sigma-Aldrich), and CD68 (sc-9139, Santa Cruz Biotechnology) antibodies. Active caspase 9 antibody (ab2324) was obtained from Abcam. Human lung tissue sections were stained with humanin (NB100-56876, Novus Biologicals) and SP-C antibodies. The corresponding secondary antibodies Alexa Fluor 594 or Alexa Fluor 488 (Invitrogen) were applied. Images were obtained using a confocal microscope (Zeiss, Jena Germany), and fluorescence intensity was quantified using ImageJ software (NIH).

### 2.6. Mitochondrial Amount and Network Analysis

Mitochondria were visualized and quantified in freshly isolated ATII cells using MitoTracker Green (Invitrogen). Mitotracker fluorescent intensity was measured using ImageJ and normalized to Hoechst 33342 intensity. Mitochondrial Network Analysis (MiNA) (https://github.com/StuartLab accessed on 14 April 2022) was used to define the morphology of mitochondrial structures quantitatively. We determined distinct morphologies: networks characterized by connected branches and individuals (punctate, rods, and large/round structures).

### 2.7. Cigarette Smoke Extract Generation

Cigarette smoke extract (CSE) was prepared using one 3R4F cigarette (Kentucky Tobacco Research & Development Center, Lexington, KY, USA) and a peristaltic pump (Manostat, Thermo Fisher Scientific, Waltham, MA, USA), as we have previously described [7]. A549 cells were treated with 20% cigarette smoke extract for 4 h, 24 h, 48 h, and 72 h.

### 2.8. ATP Measurement

ATII cells were seeded in a 96-well plate, and ATP levels were measured by CellTiter-Glo 2.0 assay (G9241, Promega, Madison, WI, USA) using a luminometer (Infinite M1000 PRO). Medium without cells was used as a blank control. Luminescence values were recorded per the manufacturer recommendations.

### 2.9. Calcium Levels Measurement

ATII cells were incubated with 2.5 μM Fluo-4, AM (F14201, Invitrogen) for 30 min, followed by an additional 10-min incubation in a dye-free medium. Flow cytometry was used for the detection of fluorescence at 488 nm.

### 2.10. Statistical Analysis

Data are expressed as means ± s.e.m from at least 3 experiments. Results were normalized to control nonsmokers. Statistically significant differences were determined by one-way ANOVA or *t*-test depending on the number of groups. A value of *p*  <  0.05 was considered significant.

## 3. Results

### 3.1. OXPHOS Gene and Protein Levels in Lung Tissue in Emphysema

**Figure 1:**
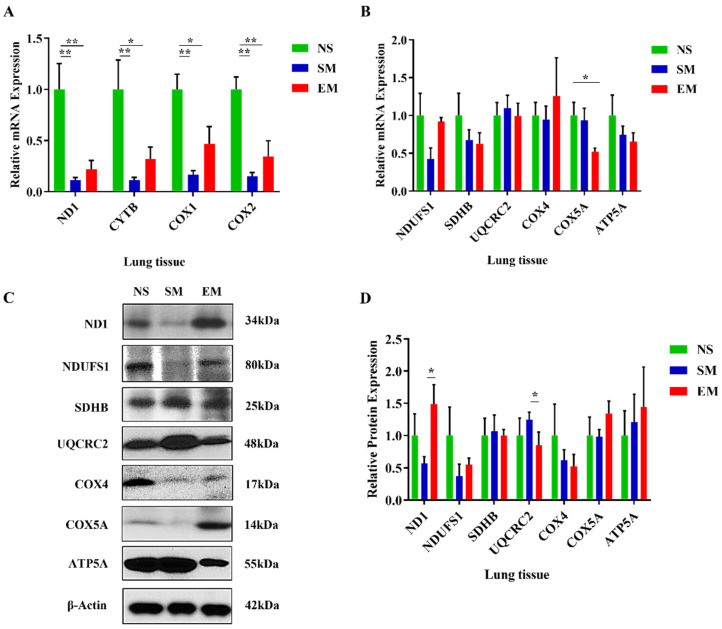
Reduced expression of mitochondria-encoded genes in lung tissue in smokers and emphysema. The expression of mitochondria-encoded (**A**) and nuclear-encoded (**B**) mitochondrial genes was determined by RT-PCR in lung tissue obtained from control nonsmokers (NS), smokers (SM), and patients with emphysema (EM). Mitochondrial protein levels were assessed by Western blotting (**C**), quantified, and normalized to β-actin expression (**D**). Data are shown as means ± s.e.m (N = 4–10 per group; * *p* < 0.05; ** *p* < 0.01).

We found a significant decrease in mitochondrial mRNA transcripts *ND1* (complex I), *CYTB* (complex III), *COX1,* and *COX2* (complex IV) in lung tissue obtained from smokers and patients with emphysema in comparison with nonsmokers by RT-PCR (Figure 1A). Our results show a significant decrease in *COX5A* mRNA expression in emphysema compared to nonsmokers (Figure 1B). We did not detect any significant differences in the levels of other analyzed genes among these groups. Next, we checked the OXPHOS protein complexes in the lung tissue (Figure 1C,D). ND1 expression was significantly increased and UQCRC2 levels were decreased in emphysema patients compared to smokers. Our results suggest a lower expression of mitochondria-encoded genes in lung tissue in emphysema compared to controls. 

Among mitochondrial mRNA transcripts, only *CYTB* expression was increased in severe emphysema compared to mild (Figure S1A). We found significantly higher UQCRC1 levels in areas with severe emphysema compared to mild (Figure S1B). However, we did not detect significant differences in OXPHOS protein complexes in the lung tissue obtained from mild and severe emphysema (Figure S1C,D). Our results can be explained by the presence of various cell types.

### 3.2. The Impairment of OXPHOS Protein Expression in ATII Cells in Emphysema

**Figure 2:**
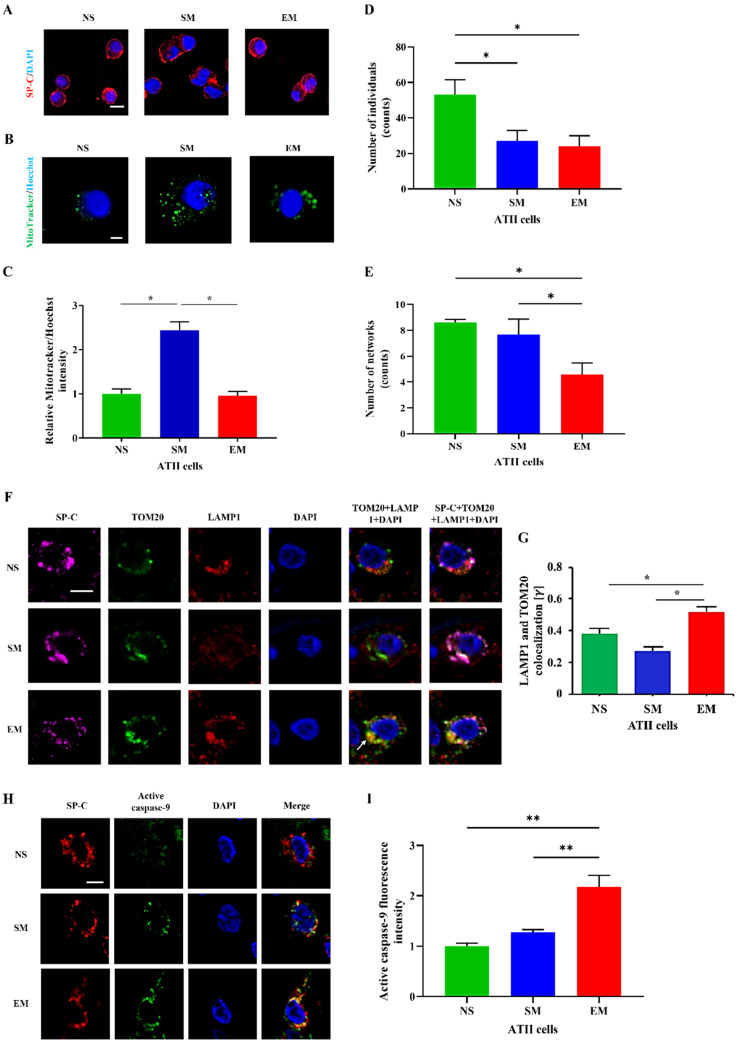
Human primary ATII cell purity and mitochondrial amount. (**A**) ATII cells were isolated from lungs obtained from nonsmokers (NS), smokers (SM), and emphysema patients (EM). Cytospins of freshly isolated ATII cells were stained using SP-C and DAPI (immunofluorescence; scale bar, 10 µm). (**B**) Mitochondrial amount was determined using MitoTracker dye and a confocal fluorescence microscope (scale bar, 5 μm). (**C**) Quantification is also shown. The number of individuals (**D**) and networks (**E**) of mitochondrial structures were quantified using MitoTracker staining. (**F**) Co-localization of mitochondrial protein TOM20 (green) with LAMP1 (red) in ATII cells using confocal microscopy (scale bar, 10 μm). (**G**) Quantification is shown. (**H**) Active caspase 9 expression (green) in ATII cells identified by SP-C antibody (red) in lung tissue sections by immunohistofluorescence (scale bar, 10 µm). (**I**) Quantification of active caspase 9 fluorescence intensity is shown. Data are shown as means ± s.e.m (N = 3–4 per group; * *p* < 0.05, ** *p* < 0.01).

The purity of freshly isolated cells was determined using SP-C, CD68, and p63 staining by immunofluorescence (Figure 2A and Figure S2). We found a significantly lower mitochondrial amount in ATII cells obtained from emphysema patients compared to smokers (Figure 2B,C). Next, we wanted to define the mitochondrial network morphology. The number of individuals (punctate, rods and structures) was decreased in the ATII cells obtained from emphysema patients and smokers compared to nonsmokers, suggesting mitochondrial fragmentation (Figure 2D). Moreover, the number of branched networks in emphysema patients was lower than in the control groups (Figure 2E). Furthermore, we found a significantly higher co-localization of TOM20 with lysosome degradation marker LAMP1 in ATII cells in emphysema compared to control organ donors (Figure 2F,G). There was no significant difference in the endoplasmic reticulum (ER) abundance in ATII cells isolated from any of the analyzed groups, indicating an effect in the mitochondria (Figure S3). Our data indicate mitochondrial dysfunction, disruption of the mitochondrial network, and mitophagy in ATII cells in emphysema which could explain the lower mitochondrial amount in ATII cells. We analyzed the active caspase 9 levels in ATII cells to determine apoptosis. We found its higher expression in emphysema patients compared to controls (Figure 2H,I), suggesting that mitophagy may trigger apoptosis. Interestingly, we also found a decreased Ki67 expression in ATII cells in smokers and patients with this disease compared to nonsmokers (Figure S4).

**Figure 3:**
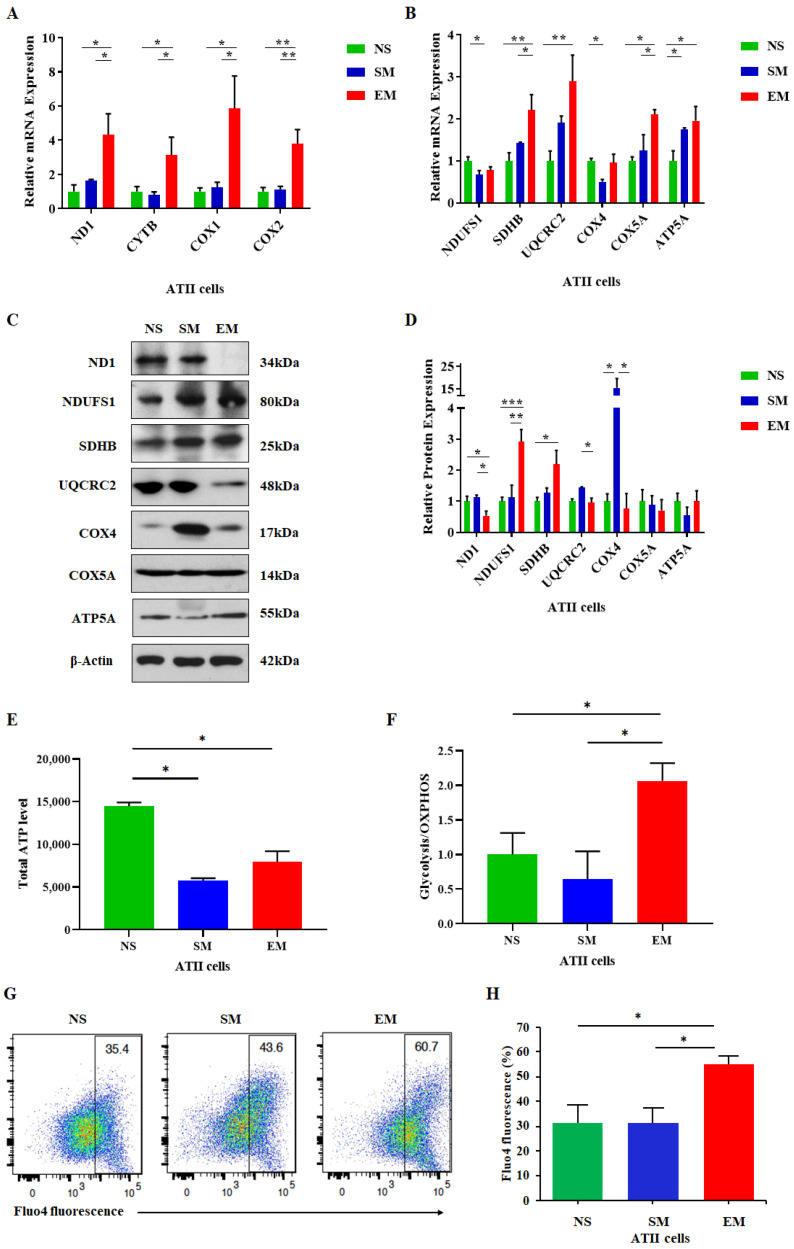
High expression of mitochondria-encoded genes in ATII cells in emphysema. Freshly isolated ATII cells from nonsmokers (NS), smokers (SM), and emphysema patients EM) were used to assess mRNA expression of mitochondria-encoded (**A**) and nuclear-encoded (**B**) mitochondrial genes. Corresponding protein levels were determined by Western blotting (**C**), quantified, and normalized to β-actin expression (**D**). (**E**) Total cellular ATP levels in ATII cells are shown. (**F**) Ratio of glycolysis/OXPHOS ATP production in ATII cells was determined. (**G**) Cellular Ca^2+^ levels using Fluo-4 by flow cytometry analysis. (**H**) Data are shown as means ± s.e.m (N = 4–14 per group; * *p* < 0.05; ** *p* < 0.01; *** *p* < 0.001).

We further analyzed the mitochondrial function in ATII cells. Unexpectedly, we detected significantly increased *ND1*, *CYTB*, *COX1*, and *COX2* mRNA levels in emphysema patients compared to nonsmokers and smokers (Figure 3A). Additionally, the expression of the nuclear-encoded genes *SDHB*, *COX5A*, and *ATP5A* was upregulated in ATII cells in this disease compared to controls (Figure 3B). *UQCRC2* mRNA expression was significantly higher in emphysema in comparison with nonsmokers. We found *NDUFS1* mRNA downregulation in smokers. ND1, UQCRC2, and COX4 levels were decreased in ATII cells in emphysema compared to smokers by Western blotting (Figure 3C,D). Interestingly, NDUSF1 protein expression was significantly increased in ATII cells in this disease compared to controls. Moreover, SDHB levels were higher in emphysema patients compared to nonsmokers. We did not detect significant changes in COX5A and ATP5A protein expression among all groups. Our results suggest that the increased mRNA expression of mitochondria- and nuclear-encoded OXPHOS subunits in ATII cells in emphysema may represent a protective response. However, the OXPHOS transcripts were less efficiently translated in this disease, resulting in mitochondrial dysfunction.

The primary function of mitochondria is ATP generation through oxidative phosphorylation. Therefore, we evaluated the overall ATP levels in ATII cells obtained from emphysema and control organ donors. We found a decreased total ATP content in this disease compared to nonsmokers (Figure 3E). Moreover, we evaluated the ratio between glycolysis and the OXPHOS ATP levels. Their ratio was higher in ATII cells obtained from emphysema patients than in controls, which indicates an increase in glycolysis (Figure 3F). Furthermore, an impaired capacity to synthesize ATP under stress conditions could affect cellular Ca^2+^. We analyzed cytosolic Ca^2+^ levels in ATII cells and detected a significant increase in emphysema compared to controls (Figure 3G,H). Together, our results suggest mitochondrial dysfunction in this disease.

### 3.3. Alteration of Mitoribosome 16S rRNA Levels in ATII Cells in Emphysema

**Figure 4:**
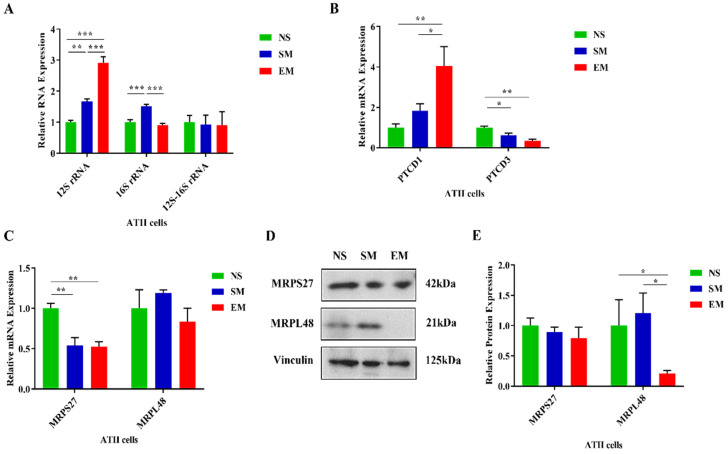
Dysregulated expression of mitoribosome components, PTCD1, and PTCD3 in ATII cells in emphysema. (**A**) The expression of mitoribosome 12S, 16S, and the junction 12S–16S rRNA was assessed using RT-PCR in ATII cells isolated from nonsmokers (NS), smokers (SM), and emphysema patients (EM). mRNA expression of PTCD1 and PTCD3 (**B**) and MRPS27 and MRPL48 (**C**) was assessed by RT-PCR. (**D**) MRPS27 and MRPL48 protein levels were evaluated by Western blotting. (**E**) Protein quantification by densitometry analysis and normalization to vinculin is shown. Data are presented as means ± s.e.m (N = 4–14 per group; * *p* < 0.05; ** *p* < 0.01; *** *p* < 0.001).

We found that 12S rRNA had a higher expression in ATII cells in emphysema patients compared to controls (Figure 4A). Unexpectedly, 16S rRNA expression was significantly lower in ATII cells in this disease in comparison with control smokers. There was no significant difference between 12S–16S rRNA junction expression among all groups, which excluded RNA transcription and processing defects. The expression of 12S rRNA was significantly reduced in lung tissue in smokers and emphysema compared to nonsmokers as determined by RT-PCR (Figure S5A). There was no difference between the 16S rRNA and 12S–16S rRNA junction levels in the analyzed groups. Together, our results indicate the post-transcriptional degradation of 16S rRNA in ATII cells in emphysema.

### 3.4. Mitoribosome Dysfunction in Emphysema

There was no difference in the *PTCD1* levels in lung tissue obtained from nonsmokers, smokers, and emphysema patients as determined by RT-PCR (Figure S5B). *PTCD3* mRNA expression was lower in this disease compared to nonsmokers. We did not detect any significant changes in PTCD1 and PTCD3 protein levels between controls and emphysema by Western blotting (Figure S5C,D). We used freshly isolated ATII cells and found a higher *PTCD1* mRNA expression in this disease in comparison with controls (Figure 4B). PTCD3 levels were decreased in smokers and individuals with emphysema compared to nonsmokers as detected by RT-PCR. However, there was no significant difference between PTCD1 and PTCD3 expression in any of the analyzed groups as determined by Western blotting (Figure S6A,B). Our results suggest that 16S rRNA stability may be independent of PTCD1 in ATII cells. Moreover, we found that *MRPS27* mRNA levels were decreased in ATII cells in smokers and emphysema patients compared to nonsmokers (Figure 4C). We did not find any differences in *MRPL48* expression among any of the analyzed groups by RT-PCR. However, MRPS27 levels were unchanged and MRPL48 expression was decreased at the protein levels in ATII cells in this disease compared to nonsmokers and smokers, as detected by Western blotting (Figure 4D,E). Moreover, we did not detect any significant changes in MRPS27 and MRPL48 levels using lung tissue obtained from nonsmokers, smokers, and emphysema patients by RT-PCR and Western blotting (Figure S7A–C).

**Figure 5:**
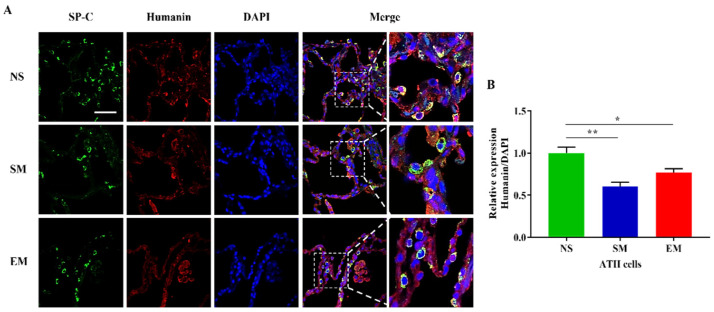
Humanin expression in ATII cells. (**A**) Lung sections were obtained from nonsmokers (NS), smokers (SM), and emphysema (EM). Staining using SP-C (green) and humanin (red) antibodies and DAPI (blue) was performed by immunofluorescence using confocal fluorescence microscopy. (**B**) Relative humanin protein expression was normalized to DAPI intensity in ATII cells identified using SP-C staining. Data are presented as means ± s.e.m (N = 4–14 per group; * *p* < 0.05; ** *p* < 0.01, scale bar, 50 µm).

We also analyzed humanin expression, which is a 16S rRNA-derived peptide, in ATII cells. Our results indicate its decreased levels in smokers and individuals with emphysema compared to nonsmokers (Figure 5A,B). Further studies are required to determine the molecular mechanism of humanin downregulation. Together, our data suggest an imbalance in rRNA and protein levels involved in the structure and the function of the mitoribosome subunits, which may result in their instability and disassembly in ATII cells in emphysema.

### 3.5. Reduced Expression of MT-RNR2-Derived Molecules in ATII Cells in Emphysema

**Figure 6:**
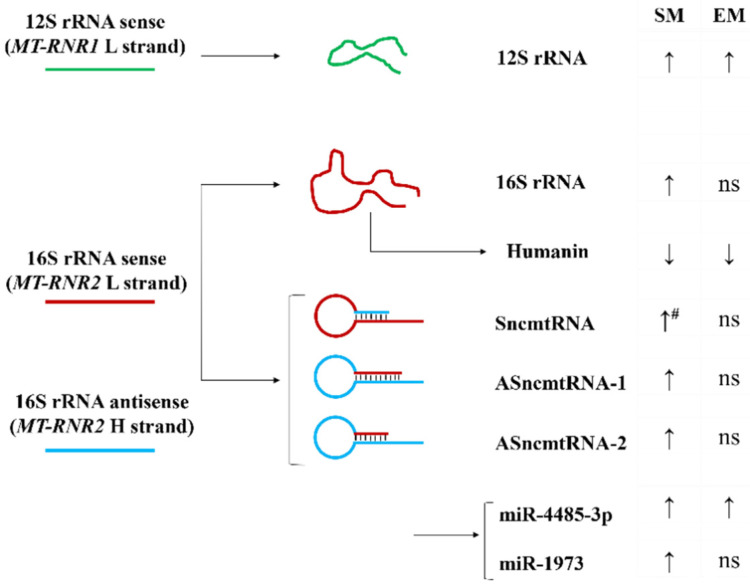
Model of MT-RNR2 derived transcripts and their expression in ATII cells. Increased (↑) and decreased (↓) RNA or protein expression in ATII cells obtained from smokers (SM) and emphysema patients (EM) compared to nonsmokers is shown (#—significantly increased expression compared to EM; ns: non-significant).

**Figure 7:**
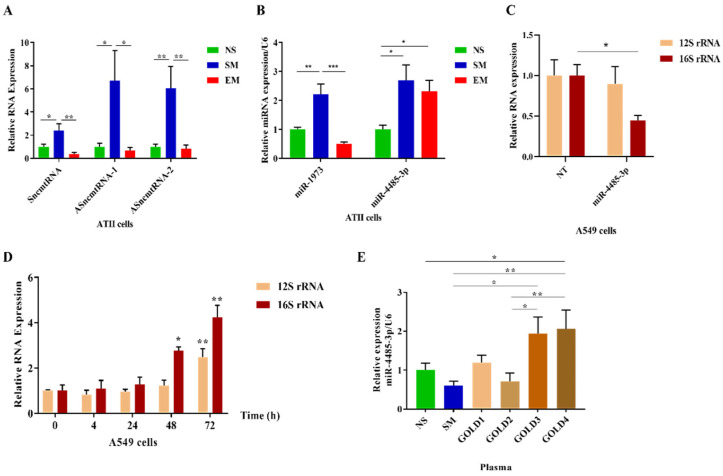
Increased miR4485-3p expression in emphysema. ATII cells were isolated from nonsmokers (NS), smokers (SM), and emphysema patients (EM). (**A**) Sense SncmtRNA and anti-sense ASncmtRNA-1 and ASncmtRNA-2 RNAs levels were normalized to GAPDH and determined by RT-PCR. (**B**) mir1973 and mir4485-3p expressions were normalized to U6 and analyzed by RT-PCR. (**C**) A549 cells were transfected with miR-4485-3p or NT for 72 h, and 12S and 16S rRNA expression was determined by RT-PCR and normalized to GAPDH (N = 3 biological replicates). (**D**) A549 cells were treated with CSE for 4 h, 24 h, 48 h, and 72 h. The expression of 12S and 16S rRNA was determined by RT-PCR and normalized to GAPDH (N = 3 biological replicates). (**E**) Plasma samples obtained from NS, SM, and COPD (GOLD1–GOLD4) were used to assess the expression of circulating mir-4485-3p (N = 4–9 per group). Results were normalized to U6 and analyzed by RT-PCR. Data are shown as means ± s.e.m (* *p* < 0.05; ** *p* < 0.01; *** *p* < 0.001).

A combination of 16S rRNA sense and anti-sense transcripts leads to the synthesis of a sense of non-coding mitochondrial RNA (SncmtRNA) and two anti-sense non-coding mitochondrial RNAs (ASncmtRNA-1 and ASncmtRNA-2) (Figure 6). First, we found that their expression was significantly decreased in ATII cells in emphysema patients compared to smokers (Figure 7A). Second, their levels were lower in nonsmokers than in smokers. These results are correlated with the 16S rRNA expression. 

Moreover, we analyzed the levels of miR1973 and miR4485-3p, which partially originate from *MT-RNR2* gene transcripts. mir1973 expression was decreased in ATII cells in emphysema compared to smokers and was lower in nonsmokers than in smokers (Figure 7B), which correlated with the 16S rRNA levels. However, miR4485-3p expression was higher in both smokers and emphysema patients in comparison with nonsmokers.

### 3.6. A miR-4485-3p Negative Feedback Loop in Emphysema

A recent study showed that mir-4485 has an interaction binding site in 16S rRNA [34], and there is an affinity between miR4485-3p and a conserved region of 16S rRNA using a BiBiServ [35]. miR-4485-3p overexpression in A549 cells significantly decreased the 16S rRNA transcript (Figure 7C). It is worth noticing that we did not observe any changes in 12S rRNA expression. Together, our results suggest 16S rRNA degradation by miR-4485-3p as a feedback loop mechanism (Figure S8). The regulation of 16S rRNA by miR-4485-3p may contribute to the stability of the mitoribosome and mitochondrial function.

A549 cells treated with 20% CSE for 48 h and 72 h had a higher expression of 16S rRNA and 12S rRNA (Figure 7D). This suggests that cigarette smoke enhances mitochondrial transcription, as observed in ATII cells isolated from smokers (Figure 4A). It may also compensate for a miR4485-3p negative feedback loop (Figure 7B).

### 3.7. Circulating miR4485-3p in Emphysema

Higher miR4485-3p expression was detected in plasma samples obtained from COPD patients (GOLD3 and GOLD4) (Table 1) in comparison to control nonsmokers and smokers (Figure 7E). Moreover, there was an increase in its levels in GOLD4 compared to GOLD2 and in GOLD3 than GOLD2. We did not detect significant differences between nonsmokers, smokers, GOLD1, or GOLD2. Additionally, miR1973 expression was analyzed in plasma obtained from patients with COPD, and we found its increased levels in GOLD1 compared to smokers (Figure S9). Together, our results suggest a potential role of circulating miR4485-3p as a biomarker of mitochondrial dysfunction in COPD.

## 4. Discussion

Here, we studied the role of mitochondrial transcription and translation machinery in ATII cells in emphysema. It has been reported that several diseases are linked to defective OXPHOS [36]. OXPHOS complexes are composed of proteins encoded by the mitochondrial and nuclear genomes. Mitochondria import nuclear-encoded proteins required for, among other processes, the electron transport chain (ETC) subunits, which rely on the balance between the transcription and translation of the mitochondrial and nuclear proteins [37,38].

Among the OXPHOS complexes, complex I plays a major role in energy metabolism, and its dysfunction is linked to more than 30% of hereditary mitochondrial encephalopathies and several human diseases [39]. We found an increase in *ND1* (complex I) mRNA expression by RT-PCR and a decrease at the protein level by Western blotting in ATII cells in emphysema. Similarly, our analysis also showed a discrepancy in UQCRC2 levels, a core component of complex III. This suggests that an inhibition of ND1 and UQCRC2 protein expression in this disease may contribute to the loss of function of complex I and complex III, respectively, leading to the dysfunctional ETC activity. This may cause an increase in mitophagy and ROS generation, which suggests decreased mitochondrial dynamics in ATII cells in emphysema, as we have previously reported [30,40]. We have shown that ATII cells isolated from patients with this disease exhibit a higher ROS production, mtDNA damage, and a low mtDNA amount [30,40]. Consistent with these data, we observed the mitochondrial network’s impairment in emphysema. Interestingly, we detected a high sequestered cytosolic Ca^2+^, which may exceed the mitochondrial Ca^2+^ retention capacity. This may lead to low ATP production by mitochondria and high glycolysis in this disease. In addition, we found the activation of caspase 9, which triggers a caspase signaling cascade to induce apoptosis. Together, our results suggest that mitophagy in ATII cells in emphysema may induce apoptosis and contribute to the disease pathophysiology.

Moreover, our results suggest an impairment of mitochondrial and nuclear transcription and translation of ETC subunits in ATII cells in emphysema. Although the levels of multiple mitochondrial transcripts were increased, we did not find a corresponding increase in the protein levels. Additionally, our data show the impairment of the nuclear/mitochondrial stoichiometry in ATII cells, since there is an increase in thelevels of nuclear proteins such as NDUFS1 and SDHB and a decrease in the expression of the mitochondrial ND1 protein. The impairment of proteasome-mediated protein degradation has been reported in emphysema [41,42], which excludes this pathway’s contribution to the observed alterations. Together, our results suggest an impairment of the translation machinery. The activation of the transcription of studied mitoribosome genes and the decreased corresponding protein levels may indicate inefficient mitochondrial translation in ATII cells in emphysema. Moreover, we found reduced mitochondrial structural protein MRPL48 levels and no significant change in MRPS27 expression. This imbalance in mitoribosome proteins’ levels may cause the disruption of its structure and subsequent steps in its assembly, leading to mitoribosome dysfunction. This is supported by a recent study showing that unassembled copies of MRPs are rapidly degraded, highlighting the importance of the mitoribosome assembly’s timing [43].

Moreover, mitochondrial rRNA expression and processing are required for mitoribosome assembly [44]. Indeed, 12S and 16S rRNAs are necessary to assemble the small and large subunits of the mitoribosome, respectively. Our results show increased 12S rRNA levels while the 16S rRNA was decreased in ATII cells isolated from emphysema patients compared to control smokers. This may also contribute to the impairment of mitoribosome assembly. Mitochondrial rRNAs are tandemly transcribed to generate a precursor, which is cleaved by RNase P and ELAC2 to separate the mature RNAs [45]. However, we did not find a significant difference in 12S–16S-rRNA junction levels among the analyzed groups, which excludes the impairment of mitochondrial gene transcription and processing in emphysema. Therefore, the decreased 16S rRNA expression in ATII cells in this disease compared to control smokers suggests its degradation during post-transcriptional events. In addition, the alteration of the 16S to 12S rRNA ratio may indicate delayed and/or impaired mitoribosome assembly. This is supported by previous observations that the impairment of this ratio may contribute to mitochondrial dysfunction [46,47,48].

The low expression of 16S rRNA in ATII cells in emphysema may also negatively affect mitoribosome protein levels. Indeed, a recent report elegantly showed that the depletion of 16S rRNA led to a decreased expression of the mitoribosome proteins of the large subunit [49]. Our results indicate a reduced expression of the MRPL48 subunit accompanying the downregulation of 16S rRNA in ATII cells in emphysema, which agrees with this study. On the other hand, we found higher 12S rRNA and *ND1* mRNA levels in ATII cells isolated from emphysema patients. However, the 16S rRNA is located between these two genes, and its expression was decreased in this disease. It has been reported that the depletion of the PTCD1 protein, which is involved in the stability and/or pseudouridylation of 16S rRNA, led to a decreased expression of 16S rRNA only—without affecting 12S rRNA expression [17,49,50,51]. We considered the possibility that the lower expression of 16S rRNA in ATII cells in emphysema may be related to its impaired processing. Therefore, we analyzed PTCD1 expression in ATII cells in this disease compared to controls. Our results indicate an unchanged PTCD1 expression among nonsmokers, smokers, and emphysema patients. This suggests that decreased 16S rRNA levels are independent of the stabilizing PTCD1 protein.

We wanted to determine whether the alternative usage of 16S rRNA may explain its low levels in ATII cells in emphysema. First, we assessed SncmtRNA, ASncmtRNA-1, and ASncmtRNA-2 expression, which are long non-coding RNAs (lncRNAs) and originate from the sense and anti-sense *MT-RNR2* gene [52,53,54,55,56]. We found their downregulation in ATII cells isolated from emphysema patients compared to control smokers. It has been reported that Argonaute and Dicer, miRNA machinery proteins, are located in the mitochondria [57,58,59], indicating that miRNA can be generated from the mitochondrial genome [60,61,62,63]. Therefore, we analyzed miRNAs that are produced by ASncmtRNA-2, namely miR4485-3p and miR1973 [54,55]. The expression pattern of miR1973 in ATII cells obtained from controls and emphysema patients was similar to 16S rRNA, suggesting a direct link between lncRNAs, *MT-RNR2* transcripts in general, and miRNA1973. miR4485-3p can be originated from either nuclear DNA chromosome 11 and/or from mitochondrial *MT-RNR2* gene transcripts. miR4485-3p had a different expression pattern than the studied *MT-RNR2*-derived rRNAs, and its levels were increased in ATII cells obtained from both smokers and emphysema patients. This suggests a negative feedback loop between miR4485-3p and 16S rRNA, which may function as a molecular switch for a 16S rRNA regulatory role in the mitoribosome. We examined whether miR4485-3p may, in turn, regulate 16S rRNA expression and act reciprocally to repress each other. Indeed, we found that A549 cells transfected with miR4485-3p mimics have decreased 16S rRNA levels. However, further investigations may determine the potential indirect effects of miR4485-3p on 16S rRNA stability.

## 5. Conclusions

In summary, we have shown mitophagy and mitochondrial dysfunction in ATII cells in emphysema. Our results also suggest the mechanisms of its progression.

## Figures and Tables

**Table 1:** Characteristics of the study cohort for analysis of circulating miRNA in plasma.

	NS	SM	COLD1	COLD2	COLD3	COLD4
**Total subjects (N)**	9	6	8	6	6	6
**Age (± SD)**	60 ± 7	71 ± 11	77 ± 7	74 ± 10	73 ± 7	64 ± 10
**Gender (% Male)**	44%	50%	63%	50%	50%	50%
**FEV_1_**	Normal	≥80%	≥80%	80% > FEV_1_ ≥ 50%	50% > FEV_1_ ≥ 30%	<30%
**FEV_1_/FVC**	Normal	≥0.7	<0.7	<0.7	<0.7	<0.7

Abbreviations: NS—nonsmokers; SM—smokers; FEV_1_-Forced expiratory volume in the first second; FVC-Forced vital capacity. Age is shown in years as median ± SD.

## Data Availability

The data supporting the findings of this study are available from the corresponding author upon request.

## Supplementary Materials

The following supporting information can be downloaded at: https://www.mdpi.com/article/10.3390/biomedicines10071497/s1, Figure S1. Expression of mitochondrial genes and proteins in areas with mild and severe emphysema in lung tissue.Figure S2. Purity of primary ATII cells, Figure S3. Expression of PDI in ATII cells. Figure S4. Ki67 expression in ATII cells. Figure S5. Expression of mitoribosome components, PTCD1 and PTCD3 in lung tissue. Figure S6. Expression of PTCD1 and PTCD3 in ATII cells. Figure S7. Expression of mitoribosome components in lung tissue. Figure S8. Model of the miR4485-3p-mediated negative feedback loop. Figure S9. Circulating miR1973 expression in plasma. Table S1. Primers used for RT-PCR.

## Abbreviations

Chronic obstructive pulmonary disease: COPD; alveolar type II cells: ATII cells; reactive oxygen species: ROS; adenosine triphosphate: ATP; oxidative phosphorylation system: OXPHOS; respiratory chain complexes: RCC; mitochondrial DNA: mtDNA; transfer RNA: tRNAs; pentatricopeptide repeat domain: PTCD; mitochondrial ribosomal protein small subunit: MRPS; mitochondrial ribosomal protein large subunit: MRPL; cytochrome C oxidase: COX; surfactant protein-C: SP-C; cigarette smoke extract: CSE; electron transport chain: ETC; sense of non-coding mitochondrial RNA: SncmtRNA; ubiquinol-cytochrome C reductase core protein 2: UQCRC2; NADH-ubiquinone oxidoreductase core subunit s1: NDUFS1.
