# Supplementary material for: Mitochondrial Ribosome Dysfunction in Human Alveolar Type II Cells in Emphysema
Source: Biomedicines. 2022 Jun 24;10(7):1497. doi: 10.3390/biomedicines10071497 (PMC9313339; doi:10.3390/biomedicines10071497)
Supplement: Supplementary file 1 [file biomedicines-10-01497-s001.zip › biomedicines-1481371- proof done-sup/biomedicines-1481371- proof done-sup.pdf]

SUPPLEMENTARY MATERIALS AND METHODS

Mitochondrial ribosome dysfunction in human alveolar type II cells in emphysema

Loukmane Karim[1,2], Chih-Ru Lin[1,2], Beata Kosmider[1,2,3], Gerard Criner[3], Nathaniel Marchetti[3], Sudhir Bolla[3], Russell Bowler[4], Karim Bahmed[2,3*]

[1] Department of Microbiology, Immunology, and Inflammation, Temple University, Philadelphia, PA 19140, USA

[2] Center for Inflammation and Lung Research, Temple University, Philadelphia, PA 19140, USA

[3] Department of Thoracic Medicine and Surgery, Temple University, Philadelphia, PA 19140, USA

[4] Department of Medicine, National Jewish Health, Denver, CO 80206, USA

*Corresponding author:
Karim Bahmed, Ph.D.
Department of Thoracic Medicine and Surgery
Center for Inflammation and Lung Research
Temple University
3500 N. Broad Street
Philadelphia, PA 19140
E-mail: karim.bahmed@temple.edu

Lung tissue cores

Lung tissue cores were obtained from areas with mild and severe emphysema of the same patient as we previously described (1). Briefly, a computed tomography (CT) scans were performed as we previously published. Tissue cores were collected from areas with mild and severe emphysema. Emphysema was quantified by the percent of the lung voxels on inspiratory CT scan with attenuation < -950 HU (Insp−950). Emphysema was considered absent in subjects with values for Insp−950 $<$ 4% in smokers, to account for the fact that the increased lung density in smokers results in a decrease in emphysema index. Severe emphysema was defined by Insp−950 $>$ 14% in smokers.

References

1. Kosmider B, Lin CR, Karim L, Tomar D, Vlasenko L, Marchetti N, Bolla S, Madesh M, Criner GJ, and Bahmed K. Mitochondrial dysfunction in human primary alveolar type II cells in emphysema. *EBioMedicine* 46: 305-316, 2019.

Table S1. Primers used for RT-PCR.

Gene		Primer's sequence (5'- 3')
ASncmtRNA-1	F	TAGGGATAACAGCGCAATCCTATT
	R	CACACCCACCCAAGAACAGG
ASncmtRNA-2	F	ACCGTGCAAAGGTAGCATAATC
	R	CAAGAACAGGGTTTGTTAGG
ATP5A	F	ATGACGACTTATCCAAACAGGC
	R	CGGGAGTGTAGGTAGAACCACT
COX1	F	GGAGCAGGAACAGGTTGAACAG
	R	GTTGTGATGAAATTGATGGC
COX2	F	CCCTTACCATCAAATCAATTGGCC
	R	ATTGTCAACGTCAAGGAGTCGC
COX4	F	CAGGGTATTTAGCCTAGTTGGC
	R	GCCGATCCATATAAGCTGGGA
COX5A	F	CATTGATGCTGCTTTGCGGG
	R	AGCCCATCCATGCGGTTTAC
CYTB	F	CTGATCCTCCAAATCACCACAG
	R	GCGCCATTGGCGTGAAGGTA
GAPDH	F	GGAGCGAGATCCCTCCAAAAT
	R	GGCTGTTGTCATACTTCTCATGG
MRPL48	F	ATGAGCGGAACCTTGGAAAAG
	R	CCACCTACAGAATAGATGGGCT
MRPS27	F	GGCTATGCACTTCTTGGGAAG
	R	GCACATCGAGCGCTTCTCTAC
ND1	F	CTACTACAACCCTTCGCTGAC
	R	GGATTGAGTAAACGGCTAGGC
NDUFS1	F	TTAGCAAATCACCCATTGGACTG
	R	CCCCTCTAAAAATCGGCTCCTA
PTCD1	F	GCCCAAATTATGGCGAGGC
	R	AGGGCTTCAACCAGCTTCC
PTCD3	F	ACAACAGACTCCATGCTGATGT
	R	AAGCGAGGGTTCTATTCCAATG
12S-16S junction	F	GAGGAGACAAGTCGTAACATGG
	R	CTATATCTATTGCGCCAGGTTTC
RNR1 (12S rRNA)	F	TAGAGGAGCCTGTTCTGTAATCGAT
	R	CGACCCTTAAGTTTCATAAGGGCTA
RNR2 (16S rRNA)	F	CCTGGTGATAGCTGGTTGTCC
	R	CAATTGGGTGTGAGGAGTTCAG
SDHB	F	GACACCAACCTCAATAAGGTCTC
	R	GGCTCAATGGATTTGTACTGTGC
SncmtRNA	F	ACAACCAGCTATCACCAG
	R	AGGTTTAGCCAAACCATT

UQCRC2	F	TTCAGCAATTTAGGAACCACCC
	R	GGTCACACTTAATTTGCCACCAA
U6	F	GCTTCGGCAGCACATATACTAAAAT
	R	CGCTTCACGAATTTGCGTGTCAT
Common Reverse	R	CGAGGAAGAAGACGGAAGAAT
miR-1973	F	ACCGTGCAAAGGTAGCAT
miR-4485-3p	F	TAACGGCCGCGGTACC
