# Supplementary material for: Mitochondrial Ribosome Dysfunction in Human Alveolar Type II Cells in Emphysema
Source: Biomedicines. 2022 Jun 24;10(7):1497. doi: 10.3390/biomedicines10071497 (PMC9313339; doi:10.3390/biomedicines10071497)
Supplement: Supplementary file 1 [file biomedicines-10-01497-s001.zip › biomedicines-1481371- proof done-sup/biomedicines-1481371- proof done-sup-1.pdf]

SUPPLEMENTARY RESULTS

Mitochondrial ribosome dysfunction in human alveolar type II cells in emphysema

Loukmane Karim[1,2], Chih-Ru Lin[1,2], Beata Kosmider[1,2,3], Gerard Criner[3], Nathaniel Marchetti[3], Sudhir Bolla[3], Russell Bowler[4], Karim Bahmed[2,3*]

[1] Department of Microbiology, Immunology, and Inflammation, Temple University, Philadelphia, PA 19140, USA

[2] Center for Inflammation and Lung Research, Temple University, Philadelphia, PA 19140, USA

[3] Department of Thoracic Medicine and Surgery, Temple University, Philadelphia, PA 19140, USA

[4] Department of Medicine, National Jewish Health, Denver, CO 80206, USA

*Corresponding author:
Karim Bahmed, Ph.D.
Department of Thoracic Medicine and Surgery
Center for Inflammation, Translational and Clinical Lung Research
Temple University
3500 N. Broad Street
Philadelphia, PA 19140
E-mail: karim.bahmed@temple.edu

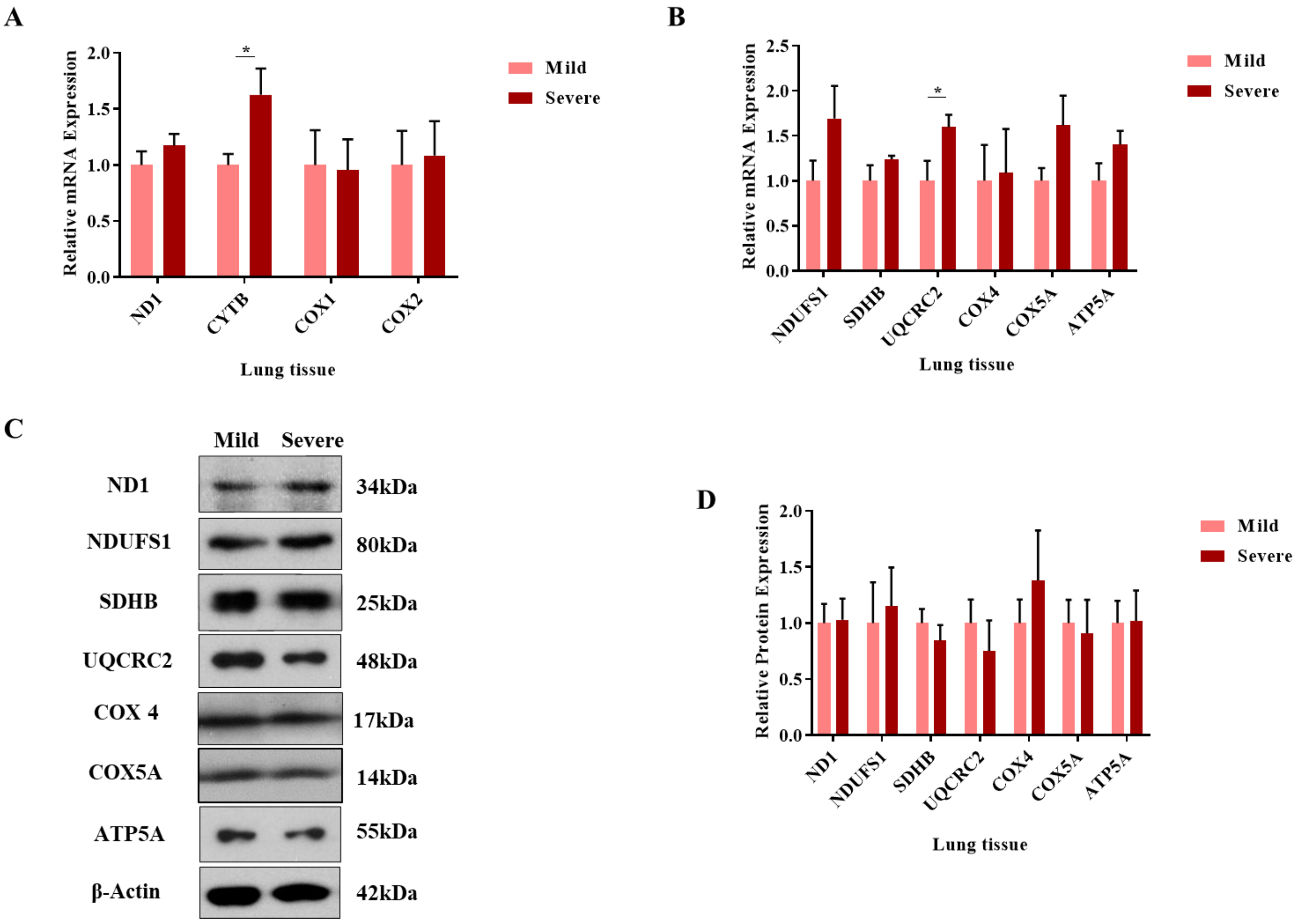

Figure S1. Expression of mitochondrial genes and proteins in areas with mild and severe emphysema in lung tissue. Expression of mitochondria-encoded (**A**) and nuclear-encoded (**B**) mitochondrial genes was analyzed by RT-PCR and normalized to GAPDH levels. Protein expression was determined by Western blotting (**C**). Data were normalized to β-actin (**D**) and are shown as means ± s.e.m (N=5-6 per group; * $P < 0.05$).

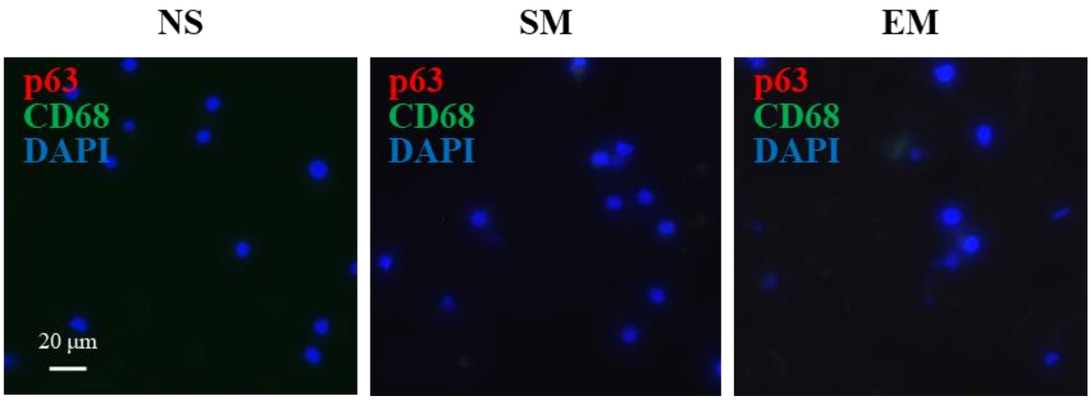

Figure S2. Purity of primary ATII cells. Cytospins of freshly isolated ATII cells were stained for p63, CD68 and DAPI using immunofluorescence (N=3 lungs per group). Scale bar, 20 μm.

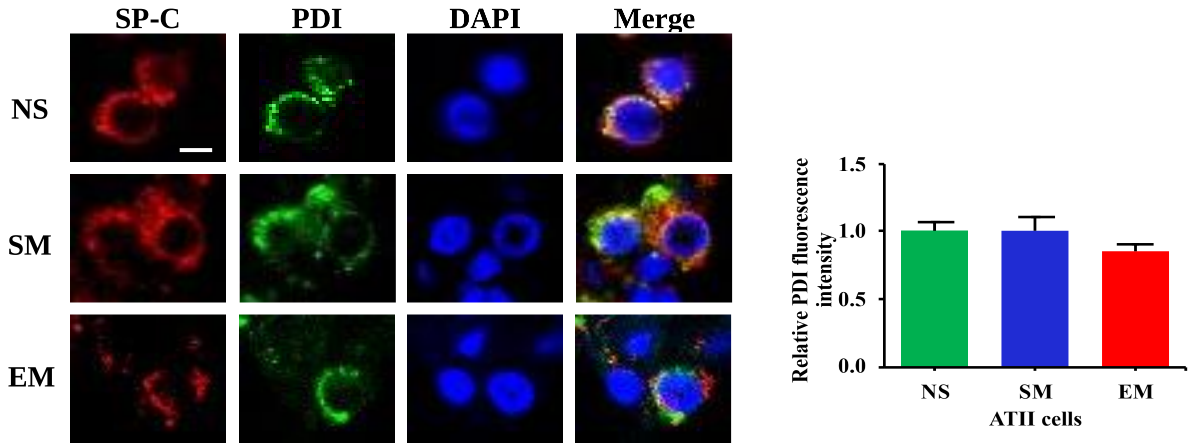

Figure S3. Expression of PDI in ATII cells. PDI fluorescence intensity was analyzed in freshly isolated ATII cells stained with SP-C by immunofluorescence (N=3 per group; scale bar, 5 μm).

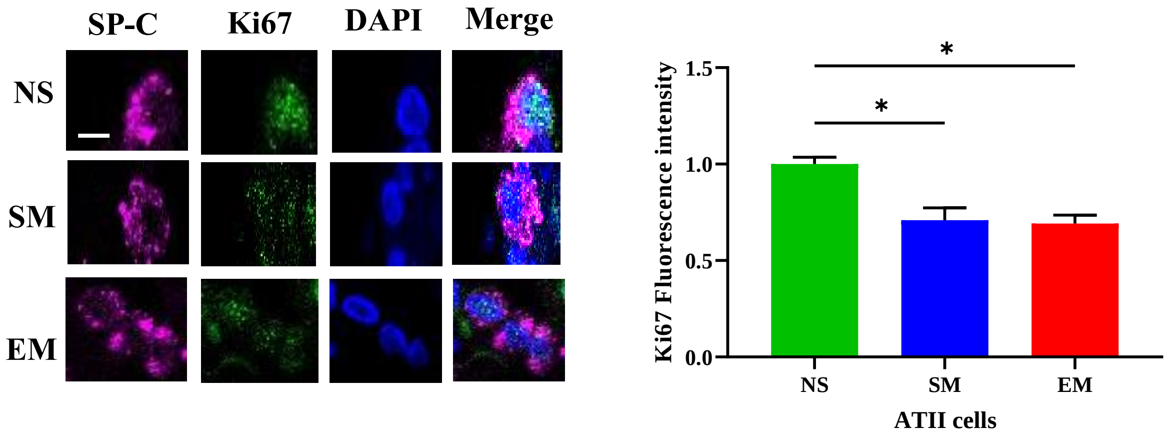

Figure S4. Ki67 expression in ATII cells. Lung tissue was obtained from nonsmokers (NS), smokers (SM), and emphysema patients (EM). ATII cells were identified using SP-C antibody by immunofluorescence). Quantification of Ki67 fluorescence intensity is shown. Data are shown as means ± s.e.m (N=3-4 per group; * $P < 0.05$; scale bar, 10 µm).

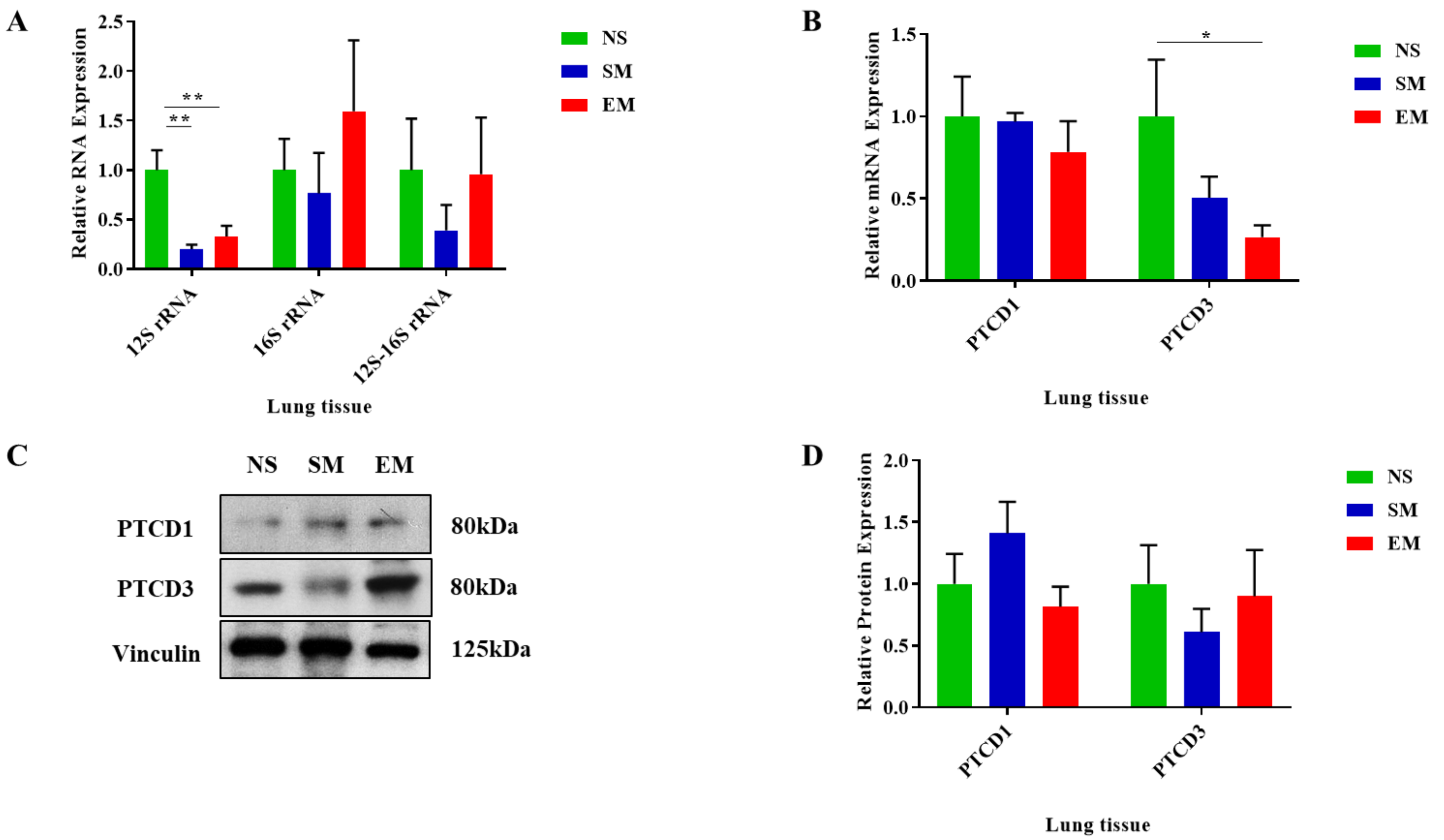

Figure S5. Expression of mitoribosome components, PTCD1 and PTCD3 in lung tissue. A- Expression of mitoribosomal 12S, 16S and the junction 12S-16S rRNA was assessed in lung tissue obtained from nonsmokers (NS), smokers (SM) and emphysema patients (EM) by RT-PCR. PTCD1 and PTCD3 mRNA and protein expression was evaluated by RT-PCR (**B**) and Western blotting (**C**), respectively. **D** – Protein quantification by densitometry analysis and normalization to vinculin is shown. Data were presented as means ± s.e.m (N=4-14 per group; * $P < 0.05$; ** $P < 0.01$).

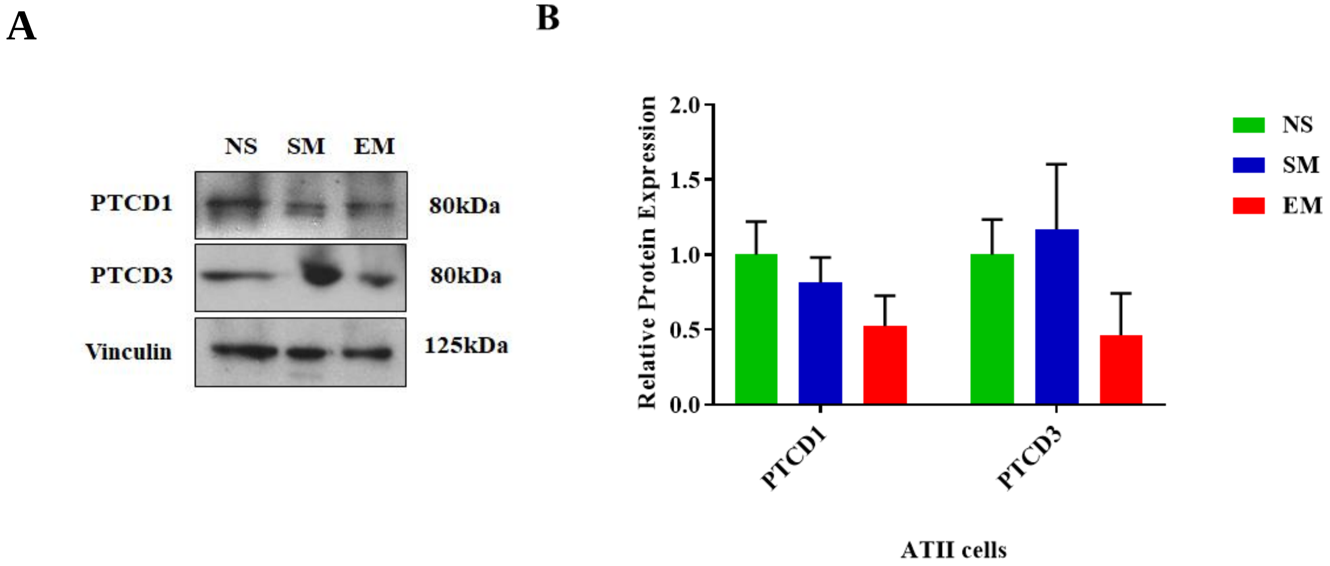

Figure S6. Expression of PTCD1 and PTCD3 in ATII cells. A - PTCD1 and PTCD3 expression was evaluated by Western blotting in ATII cells isolated from nonsmokers (NS), smokers (SM) and emphysema patients (EM). **B** – Protein quantification by densitometry analysis and normalization to vinculin is shown. Data were presented as means ± s.e.m (N=4-14 per group).

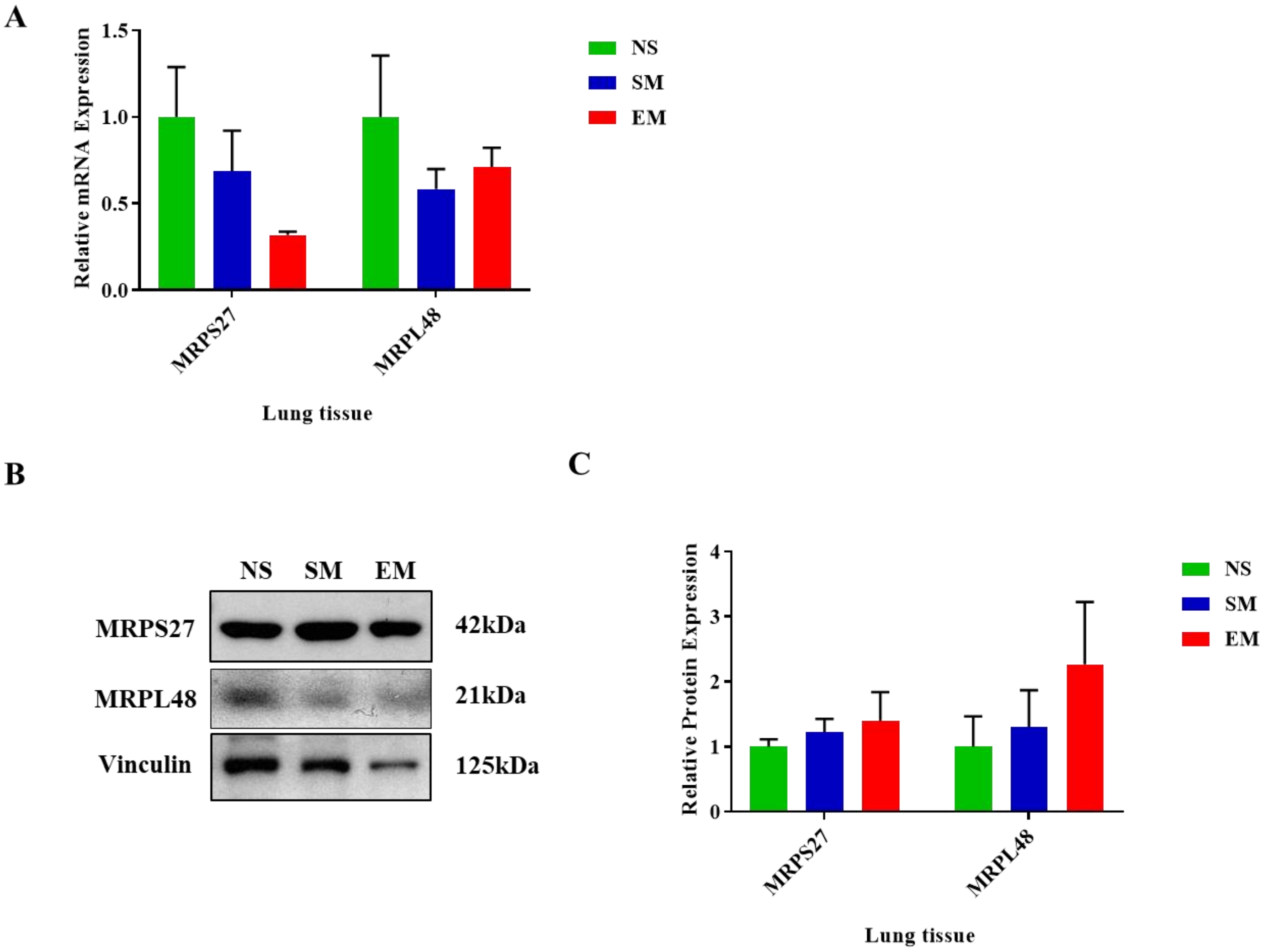

Figure S7. Expression of mitoribosome components in lung tissue. Lung tissue was obtained from nonsmokers (NS), smokers (SM) and emphysema patients (EM). MRPS27 and MRPL48 mRNA and protein expression was analyzed by RT-PCR (**A**) and Western blotting (**B**), respectively. **C** – Protein quantification by densitometry analysis and normalization to vinculin is shown. Data were presented as means ± s.e.m (N=4-14 per group).

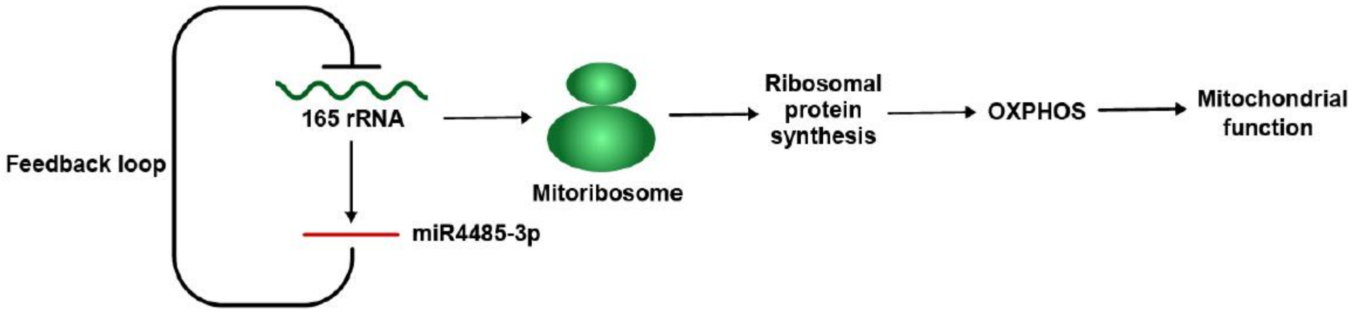

Figure S8. Model of the miR4485-3p-mediated negative feedback loop.

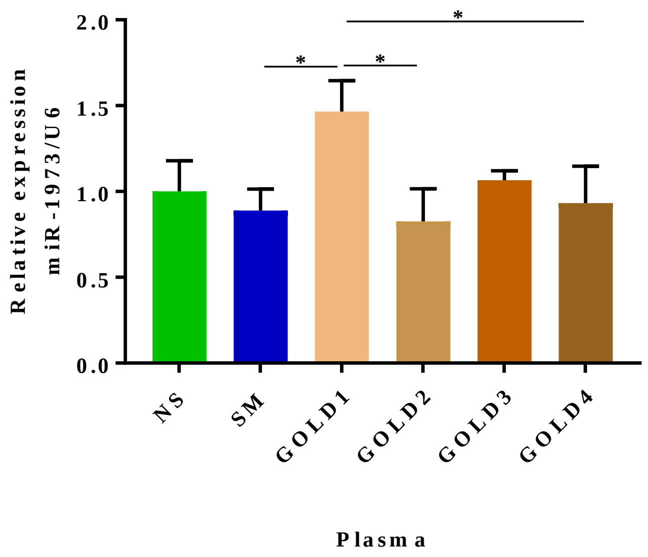

Figure S9. Circulating miR1973 expression in plasma. Plasma samples were obtained from nonsmokers (NS), smokers (SM) and COPD (GOLD1 to GOLD4). Expression of circulating miR1973 was determined by RT-PCR and results were normalized to U6. Data are shown as means ± s.e.m (N=4-9 per group; * $P < 0.05$).
